# Resource allocation and the burden of co-morbidities among patients diagnosed with chronic obstructive pulmonary disease: an observational cohort study from Danish general practice

**DOI:** 10.1186/s12913-016-1371-0

**Published:** 2016-04-06

**Authors:** Peder Ahnfeldt-Mollerup, Jesper Lykkegaard, Anders Halling, Kim Rose Olsen, Troels Kristensen

**Affiliations:** Research Unit of General Practice, Faculty of Health Sciences, University of Southern Denmark, JB Winsløws Vej 9A, DK-5000 Odense C, Denmark; Department of Health Economics, Faculty of Health Sciences, Institute of Public Health, University of Southern Denmark, JB Winsløws Vej 9B, DK-5000 Odense C, Denmark; Department of Clinical Sciences, Center for Primary Health Care Research, Lund University, Malmö, Sweden

## Abstract

**Background:**

Chronic obstructive pulmonary disease is a leading cause of mortality, and associated with increased healthcare utilization and healthcare expenditure. In several countries, morbidity-based systems have changed the way resources are allocated in general practice. In primary care, fee-for-services tariffs are often based on political negotiation rather than costing systems. The potential for comprehensive measures of patient morbidity to explain variation in negotiated expenditures for patients with chronic obstructive pulmonary disease has not previously been examined. The aim of this study is to analyze fee-for-service expenditure of patients diagnosed with chronic obstructive pulmonary disease visiting Danish general practice clinics and further to assess what proportion of fee-for-service expenditure variation was explained by patient morbidity and general practice clinic characteristics, respectively.

**Methods:**

We used patient morbidity characteristics such as diagnostic markers and multi-morbidity adjustment based on adjusted clinical groups (ACGs) and fee-for-service expenditure for a sample of primary care patients for the year 2010. Our sample included 3,973 patients in 59 general practices. We used a multi-level approach.

**Results:**

The average annual fee-for-service expenditure of caring for patients diagnosed with chronic obstructive pulmonary disease in general practice was about EUR 400 per patient. Variation in the expenditures was driven by multimorbidity characteristics up to 28 % where as characteristics such as age and gender only explained 5 %. Expenditures increased progressively with the degree of multimorbidity. In addition, expenditures were higher for patients who had diagnostic markers based on ICPC-2 (body systems and/or components such as infections and symptoms). Nevertheless, 9.8–15.4 % of the variation in expenditure was related to the clinic in which the patient was cared for.

**Conclusion:**

Patient morbidity and general practice clinic characteristics are significant patient-related fee-for-service expenditure drivers in chronic obstructive pulmonary disease care.

## Background

The association between chronic obstructive pulmonary disease (COPD) and healthcare utilization along with health care expenditure is substantial [1–4]. Overall, the increasing incidence and prevalence of COPD contribute to an increased pressure on the healthcare sector including general practice [5]. In the primary care sector it is the responsibility of general practitioners (GPs) to diagnose, treat and follow up on patients, including patients with relatively complicated and chronic diseases, e.g. COPD [6]. COPD is associated with a high degree of comorbidity from other chronic diseases [7, 8], which contributes to its high clinical and economic burden. Previous evidence has revealed specific multimorbid pairs to be associated with different levels of healthcare transitions and expenditures or costs, and identification of multi-morbidity type and linkage of information across healthcare interfaces provide opportunities for targeted intervention and delivery of cost-effective integrated care [9]. Lack of morbidity-adjusted remuneration among GPs and a dominant fee-for-service (FFS) component may lead to short GP visits focused on one problem [10]. The average patient diagnosed with COPD with comorbidities may not come back to the GP over and over again in a way that reflects their healthcare need.

The aims of this study are to analyze FFS expenditure of patients diagnosed with COPD visiting Danish GP clinics, and to assess what proportion of FFS expenditure variation was explained by patient comorbidity and GP clinic characteristics, respectively.

## Context and data

### GP setting and remuneration

In Denmark there is a national health insurance scheme covering all patients, and the majority of GP services are free of charge. GPs act as gatekeepers to the rest of the healthcare system. The GPs are compensated by the national health insurance scheme through a combination of per capita fees (30 %) and FFS (70 %). The expenditures for the health insurance scheme for FFS covers a fee for each service such as an office visit, test, clinical or diagnostic procedure, or other healthcare services. For a set of eight specified chronic diseases, including COPD, an annual checkup at the GP is expected, where the treatment plan is reevaluated, preventive measures taken into account and the patient’s psychosocial problems discussed. For this annual checkup there is a special fee, which is higher than the standard fee for surgery visit. Several countries with publicly funded general practice clinics have reoriented their remuneration systems towards a morbidity-based casemix adjustment system [11]. Denmark has yet to reorient its resource allocation system in the general practice sector towards morbidity-based casemix systems [12]. However, there seems to be a tendency or willingness to change the current system in the direction of a morbidity-based system, where a larger component is based on needs rather than the volume of visits [13].

### Classification and grouping of diagnosis

John Hopkins adjusted clinical groups (ACG) system is based on age, sex and mix of diagnoses [14, 15]. The number of visits is not included. It measures an individual’s health status and risk of health service use and has demonstrated that it is robust in its ability to measure morbidity burdens in individuals and populations [11, 16]. Denmark has recently introduced systems that could potentially allow such a system. Danish GPs have begun to use International Classification of Primary Care coding (ICPC-2) and the Danish Quality Unit of General Practice (DAK-E) has implemented automatic electronic web-based collection of ICPC-2 diagnoses for episodes of care and quality of care parameters from GP clinics [17]. These data are stored in a general practice database called DAMD. In 2008, an extended version of the ICPC-2 has been developed and implemented in the Danish general practice sector under the name ICPC-2-DK. This version is able to convert all diagnoses sent electronically from the secondary health sector (using ICD-10 – International Classification of Diseases 10) to be automatically implemented in the GP clinic IT system, ensuring a possibility for the GPs to create coherent and coordinated care across the healthcare sectors.

These new and unique data offer an opportunity to examine the relationship between current FFS expenditures and patient resource utilization, morbidity and related GP clinic characteristics. To simplify things, the ACG System Software assigns each patient into a six-level morbidity category termed a Resource Utilization Bands (RUB). The six RUBs (non-users; healthy users; low morbidity; moderate; high; and very high) are formed by categorizing the mutually exclusive ACG assignments that measure overall morbidity burden from a period of time [18].

## Ethics

The study was performed according to national and international ethical guidelines and legislation. Prior to the initiation of the study registration and approval from at the Danish Data Protection Agency was obtained. Danish legislation requires an approval from Medical Ethical Committee only for biomedical research, and as this study is based on data from registers no approval from the Medical Ethical Committee was needed.

## Methods

This is an observational, retrospective, cohort study conducted to assess healthcare resource utilization and expenditure among patients diagnosed with COPD and comorbidity. In 2010, the DAMD database contained medical and pharmacy data from 59 different GP clinics coding more than 70 % of all face-to-face encounters. These data encompassing 141 GPs and 3,973 patients diagnosed with COPD were used as the data source for this study. The medical and eligibility component files from this database were utilized, identifying patient diagnoses and procedures, and demographic characteristics. All data were fully de-identified and compliant with the legal requirements.

### Outcome measures

COPD-related resource utilization and expenditures were calculated for patients who had a registered contact with their GP during 2010 and had the diagnosis of COPD. For the diagnosis of COPD the ICPC-2 coding was used, using R79, R95 and R96 listed in chapter R (Respiratory). The examined outcomes were all cause- and COPD-related expenditures.

### Analysis

We applied a two-stage multi-level regression approach to describe and analyze the extent to which negotiated patient-level FFS expenditures are associated with COPD patients’ co-morbidity burden [18–20].

In our first stage analysis we postulated that FFS expenditure at the patient level was associated with demographics, patient morbidity measures and related GP clinic characteristics. Thus we assessed whether the FFS system prompts the GPs to provide services according to morbidity burden. Our dependent variable was defined as the total annual expenditures for the FFS remuneration of each patient diagnosed with COPD. In the first stage of our analysis we applied a fixed-effects data model that recognizes the stratification of patients within GP clinics. The GP clinic fixed effect captures the clinic specific relative expenditures (fixed effects) after allowing for differences in patient characteristics.

To explore the sensitivity of results to different morbidity and diagnostic characteristics we specified six fixed effect models with different combinations of four subsets of covariates: the age and gender of the patient, the groupings of ACGs into RUBs and a set of diagnostic markers based on ICPC-2, and number of visit to the GP.

As COPD is the index condition this diagnosis is not included in the RUBs. To measure the prevalence of diagnostic markers per patient in each of the ICPC-2 classifications, and to measure the variation explained by co-morbidity and morbidity rather than volume, we limited our analysis to the range of different diagnoses per patient (i.e., we excluded the volume of diagnosis to be able to explore the explanatory power of co-morbidity characteristics).

Gender and age have been widely applied to explain expenditure variation. Similar to the rest of the population female patients diagnosed with COPD are considered to more aware of their health [21, 22] and older patients are expected to be more expensive [23]. We included dummy variables identifying whether the patient received care for other conditions during 2010. The expectation was that the expenditure of caring for COPD patients would be higher for patients suffering from co-morbidities. For such patients FFS expenditure were expected to increase progressively with the degree and extend of co-morbidities. In the regression we excluded RUB0 as a reference group for RUB1-5.

Second stage analysis, after controlling for patient characteristics, we regressed the estimated fixed effects against a set of GP clinic characteristics to reveal the extent to which the GP-specific FFS expenditure variation was explained by observable GP clinic characteristics. We included the number of GP per clinic and number of patients enrolled per GP per clinic. This based on the supposition that if there were economies of scale [24] due to revenue economics then the annual expenditures per patient with COPD would increase as the size of the clinic in terms of number of GPs increased. We also controlled for the average physician age, the proportion of female physicians, the proportion of patient sex and patient age proportions, because FFS expenditure might be influenced by these clinic characteristics [25–27]. Finally, we explored whether the GP clinic fixed effects were associated with the proportion of co-morbidity (RUBs) and proportions of specific diagnostic markers. All analyses were performed with the use of the software package STATA version 12.0 (Stata Corp., College Station, TX, USA).

## Results

### Descriptive patient and clinic characteristics

Table 1 shows the expenditures per patient and non-diagnostic markers for the dataset, for patients diagnosed with COPD.

**Table 1:** Primary care provider and patient characteristics

	Mean	sd	p5	p50	p95
Expenditures in Euro per patient	306.70	84.63	194.25	297.31	481.31
Non-diagnostic characteristics					
Number of GPs	2.39	1.5	1.0	2.0	6.0
Age of GPs	53.57	7.67	42.00	53.67	66.00
Proportion of female GPs	0.51	0.35	0.0	0.5	1.0
Patient sex (proportion female)	0.58	0.09	0.42	0.57	0.72
Patient age (proportion 40–67)	0.56	0.11	0.40	0.56	0.76
Patient age (proportion 68+)	0.45	0.11	0.24	0.44	0.61
Number of patients	67.3				
Number of diagnoses	761.2				
Number of visits	525.0				
Number of diagnoses per visit	1.49	0.66	0.82	1.29	3.14
Number of visits per patient	7.59	1.98	5.13	7.43	11.84

P5, 5 percent percentile, P50, 50 percent percentile, P95, 95 percent percentile

**Fig. 1:**
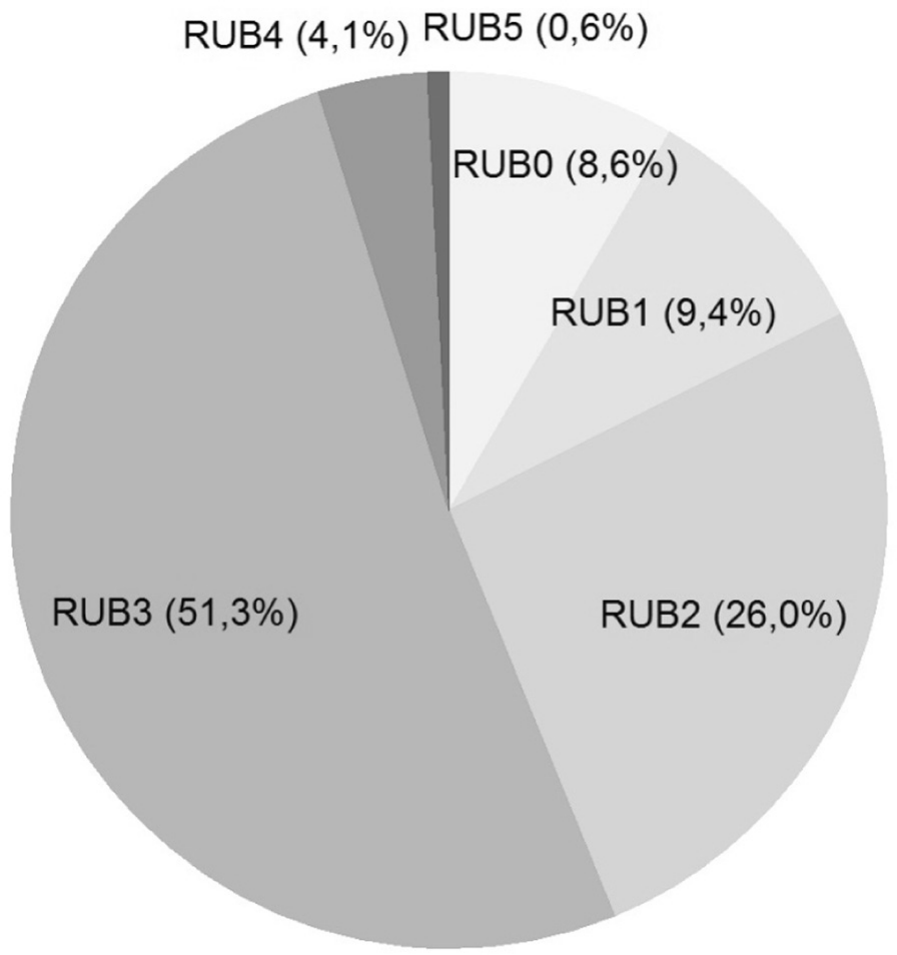
Comorbidity markers based on RUB for patients diagnosed with COPD

**Fig. 2:**
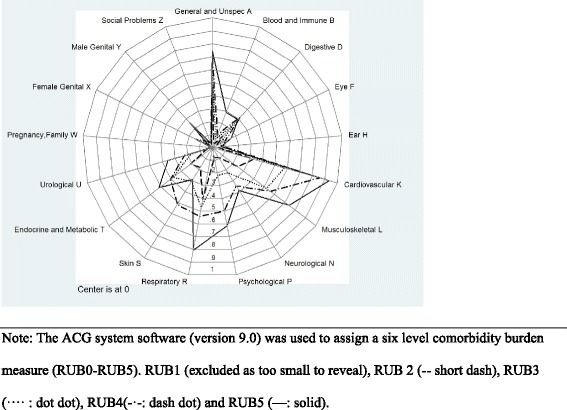
Pattern of diagnostic markers for comorbidity based on ICPC-2 chapters

**Fig. 3:**
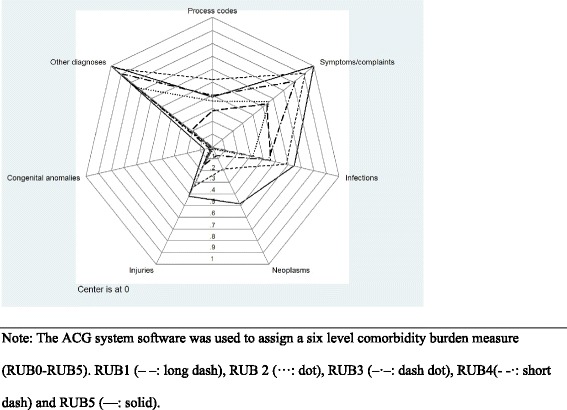
Pattern of diagnostic chapter component markers for COPD

The figures contain three subsets of the proportions of patient comorbidity characteristics for patients diagnosed with COPD: Fig. 1 shows comorbidity markers based on RUB, Fig. 2 reveals the pattern of diagnostic markers based on ICPC-2 chapters, and Fig. 3 includes diagnostic chapter component markers.

The most prevalent body system marker is ‘Cardiovascular’. In line with the classification of patients into ACGs and RUBs Fig. 2 reveals that a higher RUB-level (RUB2-RUB5) implies a higher prevalence of comorbidities in patients with COPD.

Patients with COPD at all RUB-levels (RUB1-RUB5) have received a mixture of chapter component markers besides a very small prevalence of congenital anomalies. The most prevalent COPD diagnostic markers are other diagnoses, symptoms/complaints and infections.

**Fig. 4:**
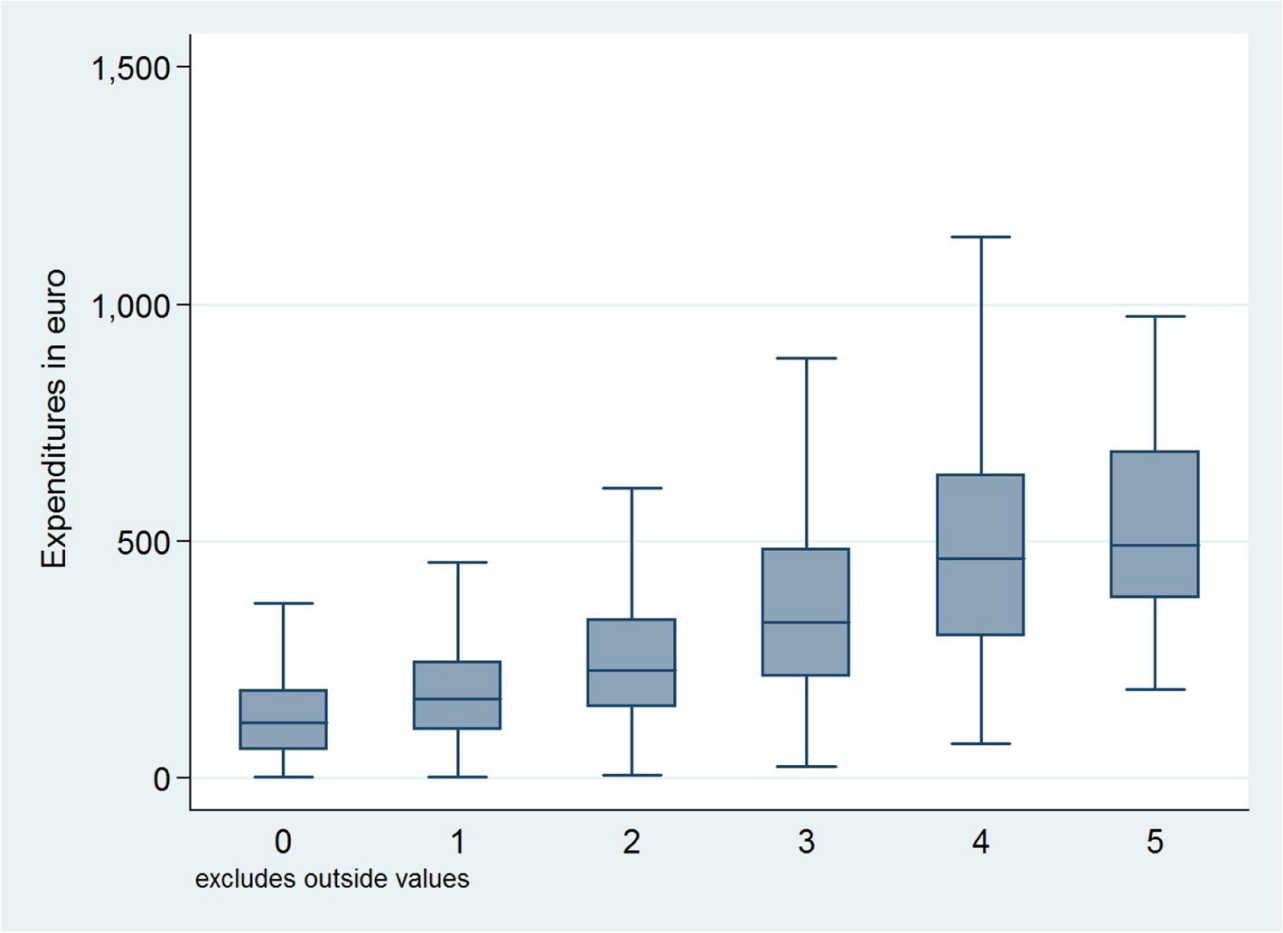
Expenditure and variation for each RUB for patients diagnosed with COPD

Figure 4 shows that patients with COPD had progressively increasing expenditure and variation with increasing level of RUB. More COPD comorbidities and other independent conditions imply higher annual expenditures. The mean (median) annual expenditures of general practice for people with multi-morbidity RUB0-5 were €139.0 (117.3), €189.6 (166.0), €272.2 (227.5) €388.5 (328.5), €531.8 (462.2) and €547.5 (492.0), respectively. This pattern is consistent with the evidence behind RUBs [18]. However, more than the descriptive statistics in Fig. 4 are needed to analyze the relative explanatory power of RUBs.

**Fig. 5:**
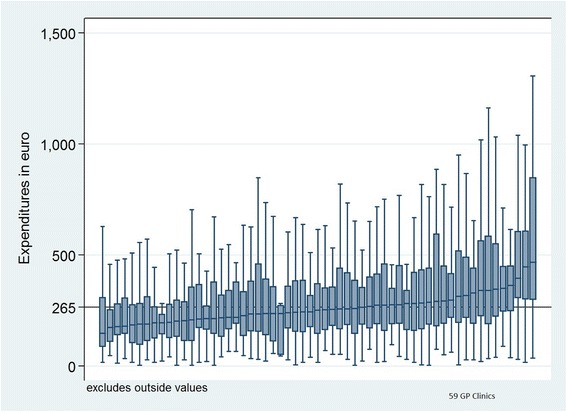
Variation between general practice clinics for expenditures for patients diagnosed with COPD

The tariff agreement on GP services and the National Health Service disbursement codes were used to calculate expenditure data and map service for each patient in 2010. Thus, we were able to identify the specific GP expenditures of patients diagnosed with COPD for 2010. Figure 5 plots these expenditures for each GP practice, thus the y-axis showing the expenditures for patients with COPD in each of the 59 GP clinics on the x-axis.

Table 2 displays the results of applying the five specifications that include different sets of COPD patient markers (demographic, multimorbidity-casemix and two sets of diagnostic characteristics). Almost all markers were significant expenditure drivers. These models are able to explain upwards 13.3–79.3 % of the variation in the FFS expenditures for primary care patients in the GP clinics, as indicated by the overall **R**2 statistics. Model 1 includes demographic case mix adjusters, and models 2–5 differ according to combinations of three sets of morbidity-based case mix adjusters and diagnostic markers: (1) RUB case mix adjusters, (2) diagnostic markers based on ICPC-2 body system chapters ‘A–Z’, and (3) diagnostic markers based on ICPC-2 chapter components. Finally, model 6 incorporates the number of visits per patient and the number of diagnoses per patient.

**Table 2:** First stage estimates: Variation in FFSE due to COPD patient characteristics and GP clinic fixed effect

	Model 1	Model 2	Model 3	Model 4	Model 5	Model 6
Demographic markers						
Age of patients with COPD	4.38^***^	3.03^***^	2.51^***^	2.85^***^	2.50^***^	1.82^***^
Proportion of female patients	25.34^**^	13.92	0.64	2.89	-1.10	0.43
RUB proportions						
RUB 1		64.92^***^	-1.29	-22.41^*^	-13.20	2.352
RUB 2		128.3^***^	6.38	9.50	-7.25	16.57
RUB 3		254.8^***^	38.42^**^	97.13^***^	23.77	27.52^*^
RUB 4		398.3^***^	104.1^***^	206.4^***^	84.11^**^	18.51
RUB 5		420.5^***^	76.03	222.5^***^	58.83	40.38
ICPC-2 chapter proportions						
A General and unspecified			53.44^***^		36.36^***^	4.38
B Blood/immune system			29.38		27.03	-0.66
D Digestive			53.68^***^		51.86^***^	2.16
F Eye			24.29		15.48	-3.41
H Ear			36.30^*^		30.57^*^	-3.12
K Cardiovascular			74.37^***^		71.82^***^	23.20^**^
L Musculoskeletal			27.26^***^		20.17^*^	-22.65^***^
N Neurological			51.12^***^		48.71^***^	9.77
P Psychological			74.71^***^		72.18^***^	35.59^***^
R Respiratory			62.57^***^		48.76^***^	7.85
S Skin			55.55^***^		39.59^***^	-5.25
T Endocrine/Metabolic and Nutritional			126.8^***^		123.5^***^	65.07^***^
U Urological			78.89^***^		70.55^***^	16.80
W Pregnancy and Family Planning			46.34		44.86	22.53
X Female Genital			17.39		12.11	-9.62
Y Male genital			16.00		15.63	-7.82
Z Social Problems			63.65^*^		67.70^*^	11.07
ICPC-2 chapter component proportions						
Process codes				53.80^***^	21.35	9.28
Symptoms/complaints				52.13^***^	11.05	1.11
Infections				76.93^***^	28.49^***^	4.56
Neoplasms				-1.04	-2.41	4.39
Injuries				57.94^***^	37.74^***^	7.41
Congenital anomalies				17.80	1.04	6.64
Other diagnoses				79.75^***^	13.32	-4.08
Volume markers						
Number of visits						21.64^***^
Number of diagnoses						4.04^**^
Constant	11.22	-84.83^***^	-70.89^***^	-85.57^***^	-69.65^**^	-73.62^***^
*N*	3973	3973	3973	3973	3973	3973
Sigma u	75.13	72.86	81.27	70.64	76.95	51.22
Sigma e	227.9	207.2	188.8	200.3	188.3	120.1
rho	0.0980	0.110	0.156	0.111	0.143	0.154
r2 o	0.0507	0.181	0.281	0.222	0.285	0.712
N g	59	59	59	59	59	59

^*^ *p* < 0.05, ^**^ *p* < 0.01, ^***^ *p* < 0.001. r2 o = Explanatory power

### Demographic markers

Model 1 shows that age and sex explained 5.1 % of the variation in FFS expenditures. For women, primary care expenditures were higher than for men in all models except for model 5.

### RUB markers

The inclusion of morbidity-based case mix adjusters (RUBs) in model 2 increased the model’s explanatory power from 5.1 to 18.1 %. Overall, FFS expenditures increased progressively with the degree of comorbidity. The coefficients for the RUBs were all related to the reference group RUB0. The expenditures for patients allocated to RUB1–5 were significantly higher than for patients allocated to RUB0. In models 3–6, the RUB coefficient decreased due to the introduction of additional morbidity characteristics (ICPC-2 chapter and component markers).

### Diagnostic markers: ICPC and Chapter component

Models 3–5 include combinations of the two sets of diagnostic markers, ‘ICPC-2 Chapter Markers’ and ‘Chapter Component Markers’, and show high levels of significant explanatory power for each of the markers. The explanatory power (**R**2 - overall) increases from 18.1 to 28.1 % (model 3), 22.2 % (model 4) and 28.5 % (model 5). The most expensive diagnostic marker for patients diagnosed with COPD seems to be endocrine/metabolic conditions followed by conditions related to urinary pathways, psychological or psychiatric conditions and cardiovascular diseases. By contrast, the chapter markers for ophthalmic diseases and ear-nose-throat diseases appear to be the least expensive among patients with COPD treated in GP clinics, but these problems are usually taken care of by specialist where no referral from GPs is needed.

### Volume markers

The inclusion of the number of visits and the number of diagnoses in model 6 increased the explanatory power from 28.5 to 71.2 % and changed the sign and magnitude of several covariates, and some became insignificant. The changed signs and magnitude of the beta coefficients in model 6 reflect the fact that some patients’ morbidity characteristics are associated with higher or lower FFS expenditure after controlling for the volume of activity. For instance, musculoskeletal condition marker leads to significantly lower expenditures per patient, and endocrine/metabolic conditions and psychological or psychiatric condition markers seem to result in larger expenditure per patient. After adjusting for the patient characteristics, the rho statistics in Table 2 indicated that GP clinic characteristics explained 9.8–15.4 % of the remaining variation in patient expenditures. Pairwise likelihood ratio tests of all combinations of the nested models (1–6) rejected all special cases in favor of model 6. In addition, model 6 was the model with the smallest AIC value.

### Variation in GP clinic fixed effects due to GP clinic characteristics (stage 2)

Table 3: In all five models, the number of GPs was significant in explaining why FFS expenditures per patient differ from one GP clinic to another. A higher number of physicians lead to more expenditures per patient. This finding suggests that larger clinics have larger expenditures per patient. It is also evident that expenditures did not vary from one clinic to another because of differences in the average physician age. A higher average physician age did not lead to more expenditure per patient. Models 6A–6E show that these results were robust to different combinations of covariates.

**Table 3:** Second stage estimations - expenditure variation due to provider characteristics

	Model 6A	Model 6B	Model 6C	Model 6D	Model 6E
Non-diagnostic markers					
No. of GPs	12.19^***^	11.71^***^	11.11^**^	12.76^*^	10.40^**^
Age of GPs	-0.30	-0.25	-0.68	-0.42	-0.66
Proportion of female GPs	3.63	10.41	3.38	17.84	2.96
Patient sex (proportion of females)		-66.25	-57.20	-59.90	-32.46
Patient age (proportion 68+)		44.46	50.03	24.84	12.75
RUB proportions					
RUB 1			-127.0	-96.43	6.311
RUB 2			103.3	96.19	208.5
RUB 3			-15.47	53.23	223.1
RUB 4			-596.2^**^	-500.5	-274.7
RUB 5			-704.9^*^	-565.4	-43.02
ICPC-2 chapter proportions					
A General and unspecified				-10.34	
B Blood/immune system				-201.6	
D Digestive				-151.1	
F Eye				-122.3	
H Ear				199.6	
K Cardiovascular				-11.75	
L Musculoskeletal				-43.20	
N Neurological				141.5	
P Psychological				-88.09	
R Respiratory				-84.22	
S Skin				68.11	
T Endocrine/Metabolic and Nutritional				47.97	
U Urological				-4.391	
W Pregnancy and Family Planning				-686.9	
X Female Genital				-23.40	
Y Male genital				-383.6	
Z Social Problems				151.2	
ICPC-2 chapter component proportions					
Process codes					11.11
Symptoms/complaints					-175.4^**^
Infections					-117.3
Neoplasms					7.581
Injuries					107.0
Congenital anomalies					-597.9
Other diagnoses					-104.7
Constant	-15.05	-1.176	40.12	60.66	93.93
N	59	59	59	59	59
R2	0.126	0.142	0.414	0.553	0.521
R2 a	0.0780	0.0608	0.291	0.164	0.323
F	4.901	3.362	3.993	2.992	6.245

^*^ *p* < 0.05, ^**^ *p* < 0.01, ^***^ *p* < 0.001

## Discussion

The average FFS expenditures for patients diagnosed with COPD in primary care were **€** 306.70. In comparison the average FFS expenditures for any primary care patient including patients with chronic conditions in 2010 were **€** 145.6 [23]. Overall we found that age, gender, RUBs and comorbidity characteristics can explain 5–28 % of the variance in FFS expenditure for the present sample of patients with COPD in general practice. This evidence, when compared with the 5.1 % obtained when only age and gender are used, indicates that comorbidity characteristics are more powerful in explaining variation in the total annual FFS for patients diagnosed with COPD in the selected GP clinics. The comorbidity casemix index, RUB, explained approximately 18 % of the variation. However, two sets of diagnostic markers indicate that expenditures are driven over and above by their RUB (morbidity measures). ICPC2-chapters (body-system & problem areas) and ICPC2-chapter component markers both increased the explanatory power of the models (model 2 - model 5). A total of 9.8–15.4 % of the variation was explained by the clinic characteristics captured in provider-specific fixed effects. When the number of visits and diagnoses were included in model 6 the explanatory power increased from 28.5 to 71.2 %.

The significance of the number of visits is consistent with the fact that the standard fee for a visit is by far the most frequently used fee, and the number of visits is by far the most powerful co-variate accounting for 42.7 % of the variation in FFS expenditure. However, the results indicate significantly higher expenditure for psychological or psychiatric and endocrine/metabolic conditions. The latter confirms that patients diagnosed with diabetes and psychiatric diseases allow for specific and supplementary fees (and lower for cardiovascular and musculoskeletal conditions).

Clinic characteristics: Different clinics have different expenditures for patients diagnosed with COPD. A higher number of GPs per clinic tend to have higher expenditure. This indicates that larger GP clinics may be able to exploit economies of scale with respect to expenditure. This may be due to better organization of the clinic and the possibility to employ clinical staff like nurses, laboratory technicians, etc. that can increase the total service and thus increase expenditure [28]. An alternative explanation is that too many cooks spoil the broth, meaning that larger clinics with more GPs patient risk to consult a different GP from time to time and the lack of continuity and personal knowledge of the patient might results in more visits to the clinic leading to an increase in expenditure. Only 9.8–15.4 % of the variation could be explained by clinic characteristics. Whereas the former result indicates some association between resource use (RUB) and FFS expenditures, the latter indicates that, even if GP clinics manage patients in different ways, the selected clinic characteristics have limited effects on FFS expenditures.

FFS is a payment model where services are unbundled and paid for separately. In health care it gives an incentive for physicians to provide more treatments, because payment is dependent on the quantity of care, rather than quality of care. Similarly, when patients are shielded from paying (cost-sharing) by health insurance coverage, they are incentivized to welcome any medical service that might do some good. FFS might discourage efficiency of integrated care, and a variety of reform efforts have been attempted, recommended, or initiated to reduce its influence (such as moving towards bundled payments and capitation). In capitation, physicians are discouraged from performing procedures, including necessary ones, because they are not paid anything extra for performing them.

Prior to potential reforms of the remuneration system, it is relevant to investigate the extent to which public resources are allocated according to morbidity status. Previous studies focusing on all types of general practice patients concluded that morbidity measures were significant patient-related fee-for-service (FFS) expenditure drivers [15–17, 29]. Morbidity characteristics explained 18–31 %, age and gender 13 % and volume of activity explained about 35 % of the resource allocation through FFS. Regardless of what chronic disease a patient might suffer from, then a recent study revealed, that multimorbidity was generally associated with greater outpatient and inpatient utilization, and, increased multisystem multimorbidity was associated with a higher outpatient share of total costs but a lower inpatient share of total costs [30]. A study of patients diagnosed with type 2 diabetes has demonstrated expenditures to increase progressively with the patients’ degree of comorbidity and being higher for patients who suffered from diagnostic markers. For diabetes type 2 patients a total of 17–25 % of the expenditure variation was explained by age, gender and patients’ comorbidity patterns, and type 2 diabetes patient comorbidity characteristics are significant patient-related FFS expenditure drivers in diabetes care [10]. The association between FFS expenditure and comorbidity burdens for COPD in our study appears to be at the same level as for diabetes type 2 and comorbidity [10]. In addition to missing morbidity adjustment, the FFS component may be too dominant in Denmark [23]. The reason for a lower expenditure variation among patients with diabetes compared to the overall GP visits not relating to a chronic disease could be that GPs actually do take care of more than one condition for each visit and are thus more efficient, this way reducing the total number of visits each patient has to the GP. Alternatively, another explanation could be that patients diagnosed with diabetes often have more diagnoses that are actually a part of the diabetic complex, which is recorded in the ICPC coding. Conditions like hypertension, dyslipidemia and obesity are all part of the diabetic complex and will thus be likely to disturb the analysis of the data.

The observed morbidity is one way of estimating needs for medical service. However, opponents may argue that the observed morbidity does not say much about how complicated the patients’ conditions are or how severe the condition is. GPs at the moment code contacts with patients to ensure an overview of each patient and thus enhance the medical quality of care. If the remuneration system is to change according to resource allocation bands and make use of diagnostic coding, the GPs’ behavior with regard to coding diagnosis may risk changing in the pursuit of a higher reimbursement, thus decreasing the value of the coding system for the purpose of quality of care.

## Strengths and weaknesses

In 2010, only a limited number of GPs (3–4 %) coded sufficiently to qualify as sentinel GP clinics. Consequently, the number of clinics for our second-stage analysis was limited. Sentinel GP clinics coding diagnoses for more than 70 % of their patients are preferred for research and monitoring. The current sample of patients was representative of all general practice patients. However, the sample of 59 sentinel general practice clinics was not representative of all Danish general practice clinics. From 2011 it has been mandatory for all GPs in Denmark to start using the sentinel data capture module and to use ICPC-2 codes for eight specific conditions: 1) COPD, 2) asthma, 3) chronic musculoskeletal disorders, 4) osteoporosis, 5) cardiovascular diseases, 6) cancer, 7) diabetes, and 8) non-psychotic psychiatric diseases. It is the plan to make use of more ICPC coding to ensure follow-up and quality of care in general practice. However, use of these ICPC codes from general practice for remuneration may lead to gaming among GPs and/or hamper the quality of the data, and thus the intended coding for quality purposes. The use of patient-level data has allowed us to explore the ways in which patient morbidity measures and related general practice clinic characteristics explain politically negotiated FFS expenditures. However from a clinical point of view the lack of adjustment for COPD severity (lung function, exacerbation history) and for socioeconomically status of the patients is a drawback, and might be able to explain further variation in the expenditures.

Patient-level analysis makes use of much more information about patients than general practice clinic-level analysis. A feature of the included sentinel general practice clinics is that they coded diagnoses on a voluntary basis in 2010. So far, no economic incentives have been agreed upon to do this. Nevertheless, it is still possible that the quality of diagnosis coding needs to be improved. Care should be taken when combining clinical diagnoses with economic incentives; the latter may very well distort the coding system. We argue that physicians in sentinel general practice clinics have been trained in coding and that they code more than 70 % of their contacts with patients. Danish sentinel general practice clinics code most chronic diseases such as diabetes and chronic obstructive pulmonary disease in a valid and reliable way. A weakness of analyzing cost drivers using retrospective FFS data as a way to guide remuneration reforms is that the dataset by definition mimics the state that needs reform. If for example the current remuneration schemes does not adequately incentivize appropriate treatment for high need patients then the dataset may not reveal the variation in need that is actually present. Therefore retrospective variation analysis should be complemented with other analysis to reform remunerations schemes. A previous study tries to select subgroups of FFS services that may be thought of as candidates to be substituted with capitation. The problem with this approach is however that there is no good guidance for how to estimate patient need [31].

## Conclusion

This study found that age, gender, and comorbidity characteristics were significant patient-related FFS expenditure drivers for patients diagnosed with COPD in general practice clinics. A higher number of GPs per clinic increases expenditure per patient diagnosed with COPD, indicating that larger general practice clinics may be able to exploit economies of scale. Further studies with more clinics and patients included are recommended. Our results, however, may indicate that there is room for improvement of the association between politically negotiated FFS expenditures and comorbidity among patients with COPD in GP clinics.
